# Calbindin content and differential vulnerability of midbrain efferent dopaminergic neurons in macaques

**DOI:** 10.3389/fnana.2014.00146

**Published:** 2014-12-03

**Authors:** Iria G. Dopeso-Reyes, Alberto J. Rico, Elvira Roda, Salvador Sierra, Diego Pignataro, Maria Lanz, Diego Sucunza, Luis Chang-Azancot, Jose L. Lanciego

**Affiliations:** ^1^Center for Applied Medical Research (CIMA), University of Navarra Medical CollegePamplona, Spain; ^2^Centro de Investigación Biomédica en Red Sobre Enfermedades Neurodegenerativas (CIBERNED)Pamplona, Spain

**Keywords:** calbindin, Parkinson’s disease, nigroextrastriatal pathway, neuronal tracers, neuroprotection, MPTP

## Abstract

Calbindin (CB) is a calcium binding protein reported to protect dopaminergic neurons from degeneration. Although a direct link between CB content and differential vulnerability of dopaminergic neurons has long been accepted, factors other than CB have also been suggested, particularly those related to the dopamine transporter. Indeed, several studies have reported that CB levels are not causally related to the differential vulnerability of dopaminergic neurons against neurotoxins. Here we have used dual stains for tyrosine hydroxylase (TH) and CB in 3 control and 3 MPTP-treated monkeys to visualize dopaminergic neurons in the ventral tegmental area (VTA) and in the dorsal and ventral tiers of the substantia nigra pars compacta (SNcd and SNcv) co-expressing TH and CB. In control animals, the highest percentages of co-localization were found in VTA (58.2%), followed by neurons located in the SNcd (34.7%). As expected, SNcv neurons lacked CB expression. In MPTP-treated animals, the percentage of CB-ir/TH-ir neurons in the VTA was similar to control monkeys (62.1%), whereas most of the few surviving neurons in the SNcd were CB-ir/TH-ir (88.6%). Next, we have elucidated the presence of CB within identified nigrostriatal and nigroextrastriatal midbrain dopaminergic projection neurons. For this purpose, two control monkeys received one injection of Fluoro-Gold into the caudate nucleus and one injection of cholera toxin (CTB) into the postcommissural putamen, whereas two more monkeys were injected with CTB into the internal division of the globus pallidus (GPi). As expected, all the nigrocaudate- and nigroputamen-projecting neurons were TH-ir, although surprisingly, all of these nigrostriatal-projecting neurons were negative for CB. Furthermore, all the nigropallidal-projecting neurons co-expressed both TH and CB. In summary, although CB-ir dopaminergic neurons seem to be less prone to MPTP-induced degeneration, our data clearly demonstrated that these neurons are not giving rise to nigrostriatal projections and indeed CB-ir/TH-ir neurons only originate nigroextrastriatal projections.

## Introduction

Parkinson’s disease (PD) is characterized by a progressive and selective loss of midbrain dopaminergic (DA) neurons. This cell loss follows a heterogeneous pattern as described in PD patients. The greatest loss of DA neurons is found in the substantia nigra pars compacta (SNc, group A9), whereas DA neurons in the ventral tegmental area (VTA, group A10) are known to be less vulnerable (German et al., 1989; Damier et al., 1999a,b; Lu et al., 2006). Within the SNc, neurons in the ventrolateral and caudal regions are more prone to degenerate than those in the rostromedial and dorsal region of the SNc. (German et al., 1989, 1992; Lu et al., 2006).

Animal models for PD showed a similar pattern of midbrain DA neurons loss (German et al., 1988, 1996; Varastet et al., 1994; Liang et al., 1996; Oiwa et al., 2003; Fitzpatrick et al., 2005). Systemic administration of MPTP to non-human primates induces a selective nigrostriatal degeneration mimicking the pattern of differential vulnerability of DA neurons observed in PD patients; the greatest loss being found in ventrolateral territories of the SNc (Schneider et al., 1987; Schneider and Dacko, 1991; Varastet et al., 1994).

It has been suggested that this selective vulnerability of midbrain DA neurons could be related with diverse differentiation routes during embryonic development (Smits et al., 2006; Smidt and Burbach, 2007), originating different DA phenotypes. Although the mechanism responsible for the preferential loss of DA neurons is still under discussion, a number of neuroprotective mechanisms have been suggested. Although several studies appointed the calcium-binding protein known as calbindin (CB; Gerfen et al., 1985, 1987; Yamada et al., 1990; Ng et al., 1996; Yuan et al., 2013) as a putative neuroprotective agent, candidates other than CB such as the vesicular monoamine transporter (VMAT2; Liu et al., 1992; Harrington et al., 1996; Miller et al., 1999; Caudle et al., 2007; Afonso-Oramas et al., 2009) have also been suggested.

The fact that CB-immunopositive neurons in the SNc are relatively preserved in patients and animal models of PD (Yamada et al., 1990; Lavoie and Parent, 1991; Ito et al., 1992; Damier et al., 1999a; Choi et al., 2008; Yuan et al., 2013), have led to the idea that CB could confer some neuroprotection to DA neurons against degeneration (Gerfen et al., 1985, 1987; Yamada et al., 1990; Ng et al., 1996; Yuan et al., 2013). CB regulates the availability of calcium ions (Ca^+2^) within the cell, thus buffering the calcium overload and thereby protecting the cell against neurotoxicity (Reisner et al., 1992). In midbrain DA neurons the Ca^+2^ channels are opened much more time than in any other cell types (Wilson and Callaway, 2000), because they show an unusual physiological phenotype; they are autonomously active showing a pacemaking activity (Grace and Bunney, 1983). The need to maintain Ca^+2^ homeostasis includes the coordination of endoplasmatic reticulum pumps, the uptake of Ca^+2^ into mitochondria and lysosome function; these Ca^+2^ pathways interact with the mitochondrial function and oxidative stress both of which appears to be involved in the pathogenesis of PD (Schapira et al., 1990; Selvaraj et al., 2009, 2012; Surmeier et al., 2011; Davey and Bolaños, 2013). Bearing in mind all these data, the CB theory hypothesized that the resilience of CB immunoreactive DA neurons in the midbrain is due to the presence of calcium binding proteins; which effectively sequester Ca^+2^ without using ATP, so CB reduces vulnerability to mitochondrial toxins and seems to confer resistance to the PD-related neurotoxic agents (German et al., 1992; Ito et al., 1992; Damier et al., 1999b; Hurley et al., 2013; Yuan et al., 2013).

Tract-tracing studies in the monkey showed that DA midbrain neurons also differ in their projection patterns (Haber and Fudge, 1997; Smith and Kieval, 2000), nigrostriatal and nigroextrastriatal projections (nigropallidal and the nigrosubthalamic) arise from different groups of midbrain DA neurons (Fallon and Moore, 1978; Lindvall and Björklund, 1979; Lavoie et al., 1989; Smith et al., 1989; Cossette et al., 1999; Hedreen, 1999; François et al., 2000; Jan et al., 2000; Smith and Kieval, 2000; Anaya-Martinez et al., 2006). The nigrostriatal projection arises from the SNc, VTA and retrorubral area (RRA): (1) the postcomissural putamen (sensorimotor striatum) is mainly targeted by DA cells located in the ventral tier of the SNc; (2) the limbic ventral striatum is innervated by DA neurons from the VTA and dorsal tier of the SNc; and (3) the caudate nucleus (associative striatum) is mainly innervated by DA neurons situated in the ventral tier of the SNc (Haber and Fudge, 1997; François et al., 2000; Smith and Kieval, 2000). The external and internal globus pallidus (GPi) as well as the subthalamic nucleus receive sparse collaterals from the nigrostriatal pathway but those nuclei also receive TH input from the nigroextrastriatal projections (Lavoie et al., 1989; Hassani et al., 1997; Cossette et al., 1999; Hedreen, 1999; François et al., 2000; Cragg et al., 2004). The nigropallidal projection is originated in the SNc and RRA (Jan et al., 2000), while the nigrosubthalamic projection arises from the SNc, VTA and RRA (Lavoie et al., 1989; François et al., 2000). The nigroextrastriatal projections also show a topographical organization similar to the one described in the striatum (François et al., 2000; Jan et al., 2000; Rommelfanger and Wichmann, 2010).

Different reports in MPTP-non-human primates have shown that the cells in the midbrain area that are able to resist chronic MPTP treatment are mostly DA neurons that express CB (CB-ir; Lavoie and Parent, 1991; German et al., 1992). The fact that the globus pallidus of MPTP-treated animals showed TH immunoreactive (TH-ir) sparing fibers (Varastet et al., 1994) suggests that the DA surviving neurons in the midbrain are involved in the nigroextrastriatal pathways instead of the nigrostriatal pathway (Parent et al., 1990; Varastet et al., 1994).

The purpose of the present study was to identify the DA midbrain neurons expressing CB in *Macaca fascicularis*, in control and MPTP-treated animals. Once the TH-ir neurons that also express CB-ir were identified in different midbrain areas, neuronal tracers were used to disclose whether these neuronal phenotypes were involved in the nigrostriatal and/or nigroextrastriatal pathways.

## Material and methods

A total of ten naïve adult male *Macaca fascicularis* primates (body weight 3.8–4.5 kg) were used in this study. Animal handling was conducted in accordance with the European Council Directive 2010/63/UE, as well as in agreement with the Society for Neuroscience Policy on the Use of Animals in Neuroscience Research. The experimental design was approved by the Ethical Committee for Animal Testing of the University of Navarra. All animals were captive-bred and supplied by R.C. Hartelust (The Netherlands).

### MPTP treatment

The dopaminergic neurotoxin 1-methyl-4-phenyl-1,2,3,6-tetrahydropyridine (MPTP; Sigma M0896) was administered intravenously to three macaques at a concentration of 0.3 mg/kg (injected weekly) until animals reached a stable parkinsonian syndrome. The severity of the MPTP-induced parkinsonism was evaluated using clinical rating scales (Kurlan et al., 1991) where the highest score was 29. The clinical features used in this scale included: facial expression (0–3), resting tremor (0–3), action or intention tremor (0–3), posture (0–2), gait (0–3), bradykinesia (0–3), balance/coordination (0–3), gross motor skills upper limb (0–3), gross motor skills lower limb (0–3), defense reaction (0–2). The MPTP-treated macaques reached a stable score between 19–23 points that was maintained over a period of 3 months of MPTP washout.

### Stereotaxic surgery, perfusion and tissue processing

Surgical anesthesia was induced by intramuscular injection of ketamine (0.5 mg/kg) and midazolam (5 mg/kg), resulting in deep anesthesia over a period of 2–3 h. Local anesthesia was implemented just before surgery by means of a 10% solution of lidocaine. As prophylaxis they received a single subcutaneous injection of metilprednisolone (10 mg/kg) and dexamethasone (0.1 mg/kg) delivered at the end of the surgical procedure and daily doses of intramuscular injection of enrofloxacin (5 mg/kg) over a period of 7 days. Analgesia was achieved with a single subcutaneous injection of carprofen (4 mg/kg–0.08 ml/Kg) delivered at the end of the surgical procedure and repeated 24 and 48 h post-surgery. After surgery, animals were kept under constant monitoring in single cages with ad libitum access to food and water.

Stereotaxic coordinates for the putamen, the caudate and GPi nuclei were taken from the atlas by Lanciego and Vázquez (2012). During surgery, target selection was assisted by ventriculography. Selected coordinates: caudate nucleus 1 mm rostral to the anterior commissure, 4.5 mm lateral to the midline and 5 mm dorsal to the intercommisural plane; putamen nucleus 1 mm caudal to the anterior commissure, 11 mm lateral to the midline and 2 mm ventral to the intercommissural plane. GPi 4.5 mm caudal to the anterior commissure, 8 mm lateral to the midline and 1.5 mm ventral to the intercommissural plane.

Two monkeys received a single pressure-injection of 5 µl of unconjugated cholera toxin subunit B (CTB, List Biological Laboratories, Campbell, CA) through a Hamilton syringe (5 mg/ml in 0.01 M phosphate buffer (PB), pH 7.5) in the dorsolateral postcommissural putamen. An additional single pressure-injection of 0.5 µl of Fluorogold (FG) through a Hamilton syringe (10% in 0.01 M PB (pH 7.5) was made in the head of the caudate nucleus. Both injections were made in the same monkey and in the same side of the brain. Two additional monkeys received a single pressure-injection of 5 µl of CTB through a Hamilton syringe (5 mg/ml in 0.01 M PB, pH 7.5) in the internal division of the globus pallidus. Tracer delivery was accomplished in pulses of 1or 0.1 µl every 2 min. Once completed, the microsyringes were left in place for 15 min before withdrawal to minimize tracer uptake through the injection tract.

Two weeks post-surgery, animals were anesthetized with an overdose of 10% chloral hydrate and perfused transcardially. The perfusates consisted of a saline Ringer solution followed by 3,000 ml of a fixative solution containing 4% paraformaldehyde and 0.1% glutaraldehyde in 0.125 M PB, pH 7.4. Perfusion was continued with 1,000 ml of a cryoprotectant solution containing 10% glycerin and 2% dimethylsulphoxide (DMSO) in 0.125 M PB, pH 7.4. Once perfusion was completed, the skull was opened, the brain removed and stored for 48 h in a cryoprotectant solution containing 20% of glycerin and 2% DMSO in 0.125 M PB, pH 7.4. Finally, frozen serial sagittal or coronal sections (40 µm-thick) were obtained on a sliding microtome and collected in 0.125 M PB cryoprotectant solution containing 20% of glycerin and 2% DMSO in 0.125 M PB, pH 7.4, as 10 series of adjacent sections.

### Detection of transported CTB and fluorogold

Immunohistochemical detection of transported CTB and FG was carried out on coronal sections. Sections were incubated with a primary antibody against CTB raised in goat (1:2000; List Biologicals, INC, 703) and rabbit antibody Anti-Fluorescent Gold (1:2000; Chemicon, AB153), diluted in a solution containing 5% of normal donkey serum (NDS) (Jackson inmunoresearch Laboratories, 017-000-121), 5% normal swine serum (Jackson immunoresearch Laboratories, 014-00-121), 0.04% triton X-100 in phosphate buffer (PBS) pH 7.4 overnight. After rinsing in PBS, sections were incubated for 2 h in a solution containing 5% of NSwS, 5% NDS, swine anti-rabbit IgG (1:50, Dako, Z0196) and donkey anti-goat (Jackson Immunoresearch, 705-065-147) diluted in PBS for 90 min; after washes sections were incubated in a solution containing 5% NDS and Goat PAP (1:600 Sigma, P1901) and afterwards washed in PBS and visualized in brown with DAB (Sigma, D5637). Section were washed in PBS and incubated in a solution containing 5% NSwS and Rabbit PAP (1:50 Dako, Z0113 diluted in PBS and visualized using Vector VIP Peroxidase (HRP) Substrate Kit (Vector laboratories, SK-4600). Sections were mounted on gelatin-coated glass slides, dried at RT and coverslip with Dpex (VWR International).

### Detection of transported CTB and FG combined with TH and CB immunofluorescence

The following primary antibodies were used in double inmunofluorescence: (1) a mouse anti-calbindin-D-28K (1:2000, Sigma, C9848); (2) a goat anti- TH (1:50, Santa Cruz, sc-7847); (3) a rabbit anti-Fluorescent Gold Antibody (1:2000, Chemicon, AB153); and (4) a rabbit anti-CTB (1:2000, Genway, 18-272-195906).

In the present study we have used the following secondary antibodies: Alexa Fluor^®^ 633 Donkey Anti-Mouse IgG (1:200, Molecular Probes A 21082); Alexa Fluor^®^ 546 donkey anti-goat IgG (1:200, Molecular Probes A 11056), Alexa Fluor^®^ 488 donkey anti-rabbit IgG (1:200, Molecular Probes A 21206), biotinylated Donkey anti-rabbit IgG (1:600, Jackson Laboratories, 711-066-152) and biotinylated Donkey anti-mouse IgG (1:600, Jackson Laboratories, 715-066-150).

For single and triple immunohistochemistry, free-floating sections were incubated in a blocking solution containing 5% of NDS and 0.04% triton X-100 in PBS pH 7.4 for an hour. After that, sections were incubated overnight at room temperature with the appropriate primary antibody or mix of antibodies, diluted in a solution of 5% NDS, 0.04% triton X-100 in PBS.

For single immunohistochemistry, after rinsing in PBS, sections were sequentially incubated with the appropriate secondary antibody diluted in a solution containing 5% of NDS in PBS for 2 h and afterwards washed in PBS. Finally sections were incubated in a solution of HRP-conjugated streptavidin (1:5000, Sigma, E2886) diluted in PBS for 90 min and visualized in brown with DAB (Sigma, D5637).

For triple immunofluorescence, after rinsing with PBS sections were incubated with the appropriated fluorescent secondary antibodies and diluted in a solution containing 5% NDS in PBS for 2 h.

Finally, sections were rinsed in PBS and mounted on SuperFrost Ultra Plus^®^ slides, dried at RT and coverslip with Dpex (VWR International).

### Sections sampled and quantification of TH^+^/CB^+^ co-localization

One series of sections from three control and three MPTP-treated macaques were used for quantifying TH+/CB+ co-localization in double-stained material. Briefly, three equally-spaced coronal sections through the mesencephalon (1.5 mm apart from each other) were entirely scanned under the confocal microscope. One section was taken at the level of the exit of the third cranial nerve to properly elucidate the boundaries between the SNc and VTA. Next, two more rostral and caudal coronal sections (1.5 mm rostral and caudal to the exit of the third cranial nerve, respectively) were chosen for counting purposes. The percentages of co-localization were gathered from 2,615 +/− 640 TH+ neurons in control macaques and from 1,112 +/− 145 TH+ neurons in MPTP-treated animals.

### Confocal visualization settings

Stained samples (immunofluorescence and PLA) were inspected under a Zeiss 510 Meta confocal laser-scanning microscope (CLSM). To ensure appropriate visualization of the labeled elements and to avoid false positive results, the emission from the argon laser at 488 nm was filtered through a band pass filter of 505–530 nm and color-coded in green. The emission following excitation with the helium laser at 543 nm was filtered through a band pass filter of 560–615 nm and color coded in red. Finally, a long-pass filter of 650 nm was used to visualize the emission from the helium laser at 633 nm and color coded in pale blue.

## Results

### TH and CB expression in control and MPTP-treated monkeys

**Figure 1:**
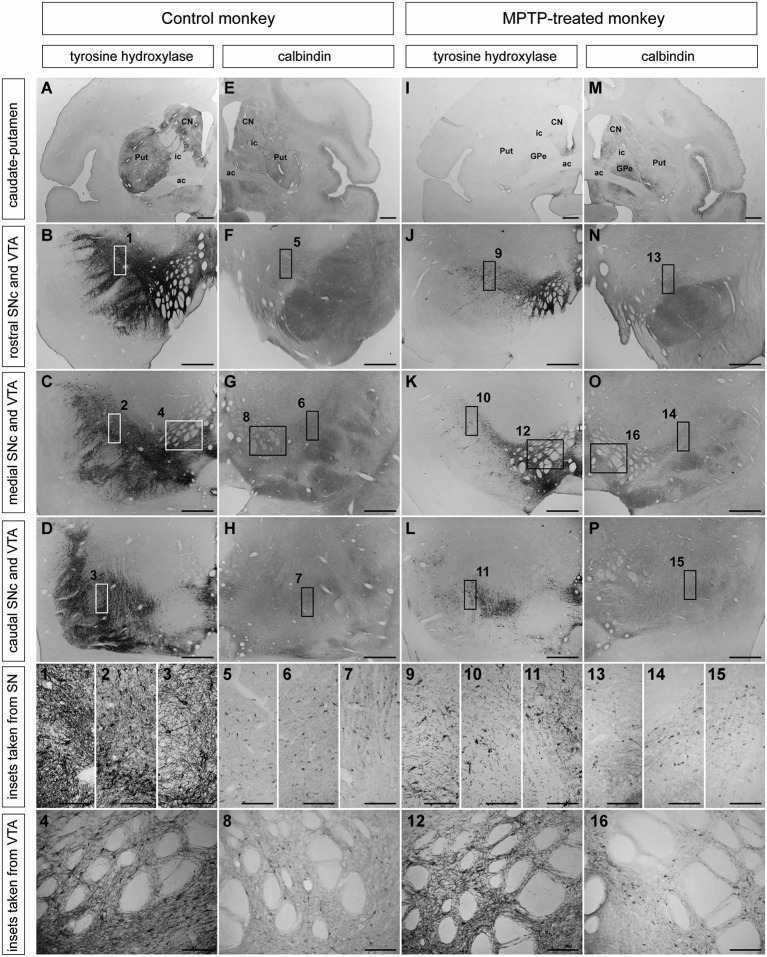
**Distribution patterns of TH and CB immunoreactivity in caudate and putamen, SNc and VTA in control and MPTP-treated monkeys**. **(A–D)** TH immunohistochemistry in control monkeys in striatum **(A)**, rostral, medial, and caudal SNc **(B–D)** and VTA. **(1–4)** Insets taken at higher magnification of rostral **(1)**, medial **(2)**, and caudal **(3)** SNc and VTA **(4)** showing TH-ir profiles. **(E–H)** CB immunoreactivity in striatum **(E)**, rostral, caudal, and medial **(F–H)** SNc and VTA. **(5–8)** Higher magnification insets of CB-ir cells in rostral **(5)**, medial **(6)** and caudal **(7)** SNc, and VTA **(8)**. **(I–L)** Extent of MPTP-induced dopaminergic depletion. The levels of TH in the striatum and SN that typically characterizes a control primate **(A–D)** are clearly reduced in the MPTP-treated monkeys **(I–L)**. **(9–12)** Higher magnification insets of TH-ir cells in the rostral **(9)**, medial **(10)** and caudal **(11)** SNc as well as in the VTA **(12)**, showing a reduction of the TH immunoreactivity compared with control monkeys **(1–4)**. **(M–P)** calbindin immunohistochemistry in striatum **(M)**, rostral, medial, and caudal SNc **(N–P)** and VTA. **(13–16)** Insets taken a higher magnification from rostral **(13)**, medial **(14)**, and caudal SNc **(15)** and VTA **(16)** showing the presence of CB-ir cells in these areas. Unlike the TH expression, CB immunoreactivity did not changed between control and MPTP-treated monkeys. Scale bars: 1,000 µm in panels **(A, E, I and M)**; 250 µm in panels **(B–D, F–H, J–L and N–P)**; 25 µm in panels **(1–16)**. Abbreviations: substantia nigra, pars compacta (SNc), ventral tegmental area (VTA), putamen (Put), caudate nucleus (CN), external division of the globus pallidus (GPe), internal capsule (ic), anterior commissure (ac).

All three monkeys intoxicated with MPTP developed a stable parkinsonian syndrome between 5 and 8 months after the initiation of MPTP administration, scoring between 19–23 points in the accumulative Kurlan scale (Kurlan et al., 1991). The immunohistochemistry for TH confirmed the extension of the nigrostriatal damage induced in the three monkeys treated with MPTP, when compared with the TH stain in the control monkeys (Figure 1).

Overall, the pattern of degeneration in the ventral midbrain and striatum was similar for the three monkeys. As expected, the caudate and putamen of control monkeys showed numerous and intense TH-ir (Figure 1A), whereas MPTP-treated monkeys showed a marked reduction in TH density in both caudate and putamen nuclei (Figure 1I). In control monkeys, TH-ir neurons were easily noticed in the dorsal and ventral tiers of the SNc (Figures 1B–D). MPTP treatment induced a severe loss of dopaminergic neurons, although few TH-ir cells within the dorsal and ventral tiers of the SNc were still visible. (Figures 1J–L). By contrast, only very small differences in TH immunoractivity were observed in the VTA when comparing control and MPTP-treated monkeys (Figures 1C,K).

CB immunohistochemistry in control and MPTP-treated monkeys was carried out using sections adjacent to those stained for TH. The striatum, SNc (rostral, medial and caudal), and VTA showed similar levels of CB-immunoreactivity (CB-ir) in both control and MPTP-treated monkeys (Figure 1).

**Figure 2:**
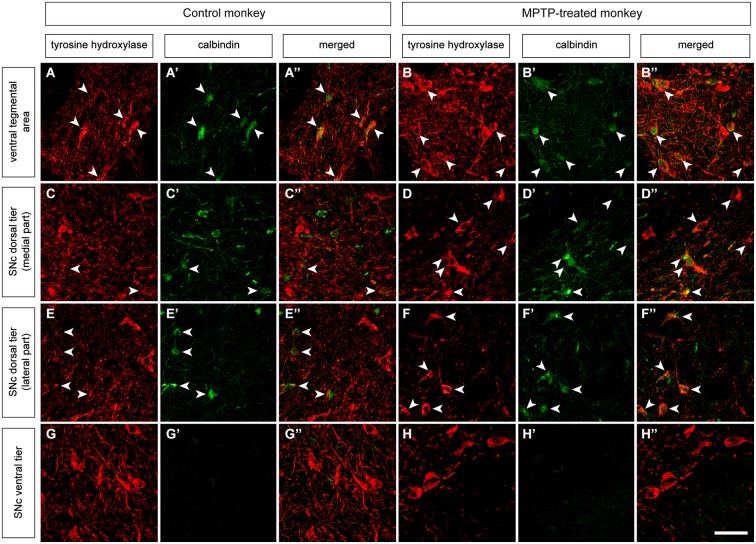
**Double immunofluorescent detection of TH and CB in VTA and SNc in control and MPTP-treated monkeys**. At the level of the VTA in control macaques **(A–A”)**, 58.2% of the CB-ir neurons are also TH-ir, and 62.1% in MPTP-treated animals **(B–B”)**. When considering the dorsal tier of the SNc (SNcd, medial and lateral territories) of control animals **(C–C” and E–E”)** the percentage of co-localization is estimated as 34.7% (arrowheads). By contrast, in MPTP-treated macaques and besides a substantial loss of TH-ir neurons, most of the surviving dopaminergic neurons expressed CB (arrowheads in **D–D”** and **F–F”**). The percentage of TH-ir/CB-ir neurons increased up to 88.6%. In other words, dopaminergic neurons from the SNcd containing CB are less prone to degenerate following MPTP insult. At the level of the ventral tier of the SNc (SNcv) none of the TH+ neurons expressed CB in control animals **(G–G”)**. Panels **(H–H”)** are taken from the boundaries between SNcd and SNcv, since most -if not all- of the TH-ir neurons in the SNcv degenerated following MPTP treatment because of the lack of CB in these cells. Scale bar is 25 µm in all panels.

Double-labeling stains for TH and CB were carried out in the VTA and SNc in control and MPTP-treated monkeys (Figure 2). In control animals, and in keeping with previous studies, the highest percentage of co-localization was found in the VTA, where 58.2% of TH-ir neurons also showed immunoreactivity for CB (Figure 2). At the level of the SNc, 34.7% of TH-ir neurons from the dorsal tier (SNcd, medial and lateral territories) also expressed CB-ir, whereas TH-ir neurons in the ventral tier did not colocalized with CB. As expected, TH-ir neurons located in the ventral tier (SNcv) lacked CB immunoreactivity (Figure 2). MPTP-treated macaques showed a similar percentage of TH/CB colocalization in the VTA (62.1%). Moreover, most of the surviving TH-ir neurons (88.6%) in the SNcd also expressed CB (Figure 2). Similar to what was found in the SNcv for control macaques, TH-ir neurons in the SNcv did not expressed CB in MPTP-treated animals (Figure 2).

### Distribution of nigrostriatal-projecting neurons within the SNc as seen with retrograde tracers

**Figure 3:**
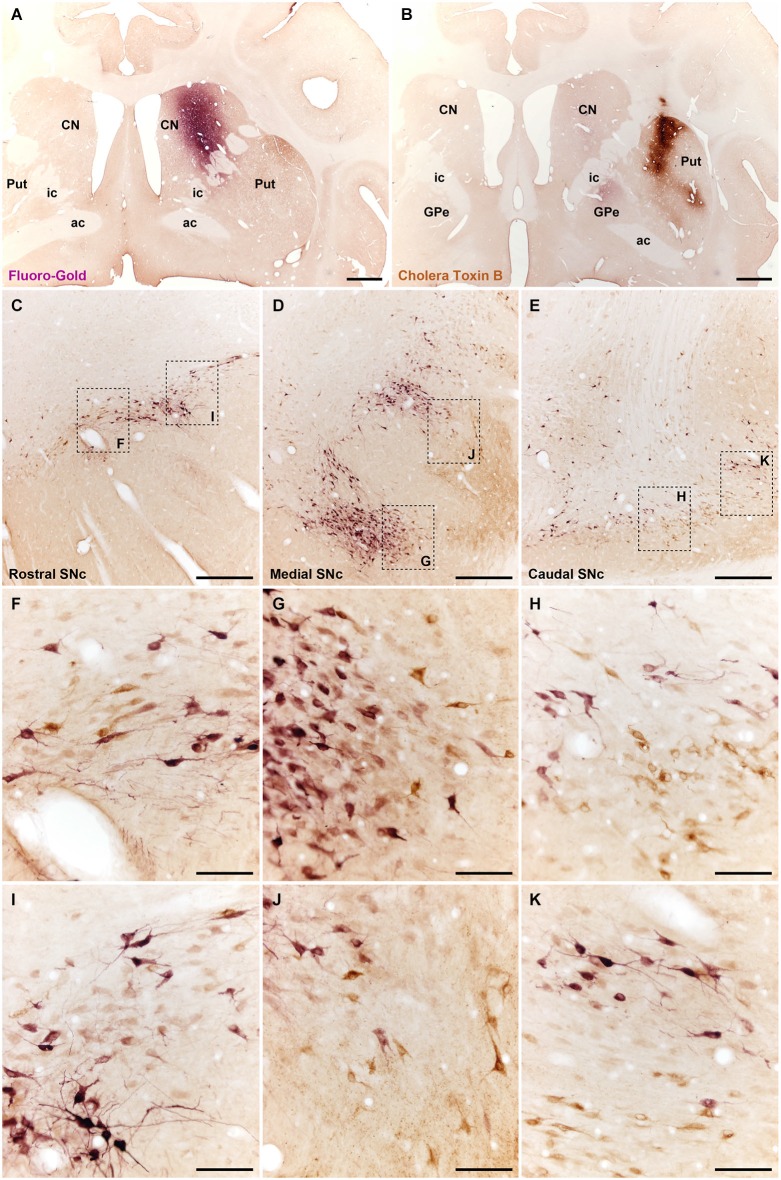
**Different types of nigrostriatal-projecting neurons identified following the delivery of retrograde tracers in the head of the caudate nucleus (A; injection of FG) and in dorsolateral territories of the postcommissural putamen (B; CTB deposit)**. Both injections are restricted to the targeted area, without any noticeable tracer leakage through the injection tract. **(C–E)** coronal sections through the substantia nigra taken from rostral **(C)**, medial **(D)** and caudal **(E)** levels. Neurons innervating either the caudate nucleus (purple-stained; labeled with FG) or the putamen (brown-stained; labeled with CTB) are distributed in clusters containing both subtypes of neurons intermingled with each other. **(F–K)** Insets taken from **(C–E)** at higher magnification to better appreciate the cellular composition of the clusters containing projection neurons. It is worth noting that double-labeled cells, e.g., neurons innervating both the caudate and the putamen were never noticed. Scale bar is 2,000 µm in panels **(A)** and **(B)**; 500 µm in panels **(C–E)**, and 100 µm in panels **(F–K)**. Abbreviations: putamen (Put), caudate nucleus (CN), external division of the globus pallidus (GPe), internal capsule (ic), anterior commissure (ac).

Control animals received two injections of the retrograde tracers FG and CTB into the caudate and putamen nuclei, respectively. Tracer leakage through the injection tracts was not observed in any of the injected animals (Figures 3A,B). In all cases, CTB- and FG -labeled neurons were found throughout rostral, medial and caudal territories of the ipsilateral SNc (Figures 3C–E), and in the ipsilateral VTA. Labeled neurons were distributed in clusters across the entire rostrocaudal extent of the SNc. Although both types of projection neurons were intermingled with each other in all clusters, double-labeled neurons were never observed (Figures 3F–K). Few scattered cells labeled with either CTB or FG were also found in the VTA.

### Expression of TH and CB in identified nigrostriatal dopaminergic neurons

**Figure 4:**
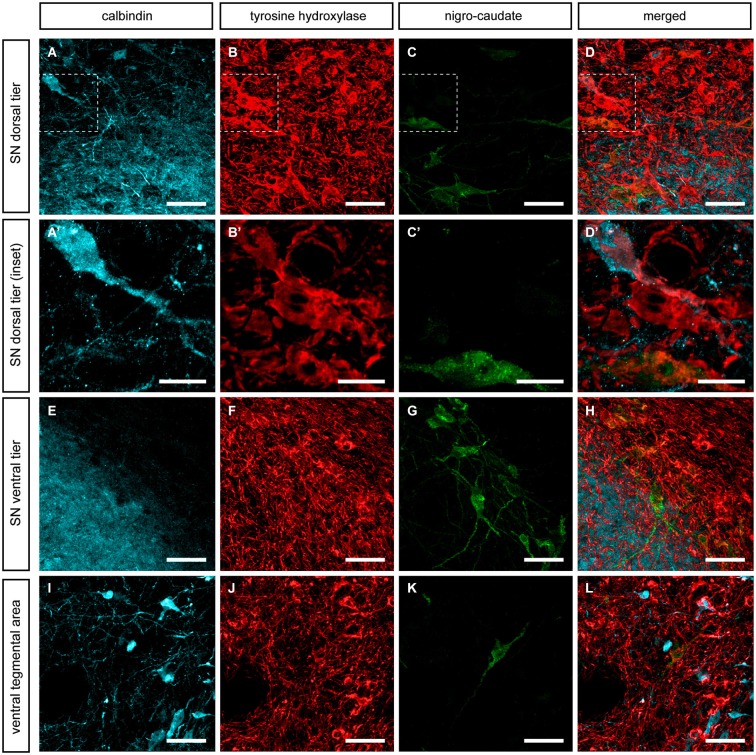
**Nigrostriatal neurons innervating the caudate nucleus**. Following the delivery of FG in the head of the caudate nucleus, retrogradely-labeled neurons (green channel) were found in the SNcd **(C)**, in the SNcv **(G)** and to a lesser extent in the VTA **(K)**. All these FG-ir neurons are TH-ir and negative for CB. **(A’–C’)** Insets taken from **(A–D)** showing one FG+/TH+ neuron, one CB-ir/TH-ir neuron as well as another neuron single-stained for TH only. It is worth noting that FG-ir/TH-ir/CB-ir neurons were never seen. Scale bar is 25 µm for panels **(A–D, E–H and I–L)** and 5 µm in panels **(A’–D’)**.

**Figure 5:**
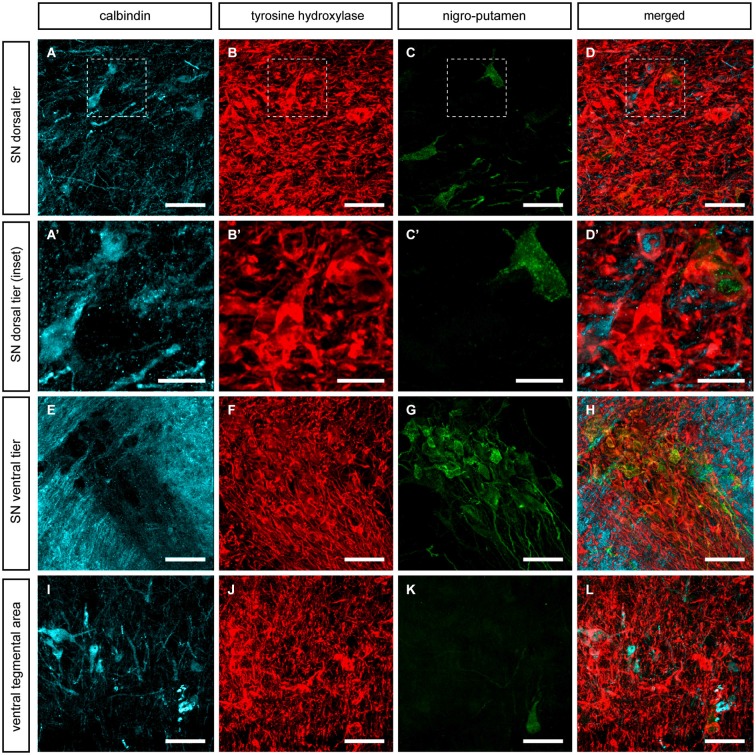
**Nigrostriatal neurons innervating the putamen nucleus**. Following the delivery of CTB in dorsolateral territories of the postcommissural putamen, retrogradely-labeled neurons (green channel) were found in the SNcd **(C)**, in the SNcv **(G)** and to a lesser extent in the VTA **(K)**. All these CTB-ir neurons are TH-ir and negative for CB. **(A’–C’)** Insets taken from **(A–D)** showing one CTB-ir/TH-ir neuron, two CB-ir/TH-ir neurons as well as few more neurons single-stained for TH only. It is worth noting that FG-ir/TH-ir/CB-ir neurons were never seen. Scale bar is 25 µm for panels **(A–D, E–H and I–L)** and 5 µm in panels **(A’–D’)**.

To elucidate the presence of CB-ir within identified nigrostriatal-projecting midbrain dopaminergic neurons, triple immunofluoresce stains detecting TH, CB and FG or CTB were conducted in control animals (Figures 4, 5). Following the deposit of FG in the caudate nucleus, labeled neurons were found in both the dorsal and ventral tiers of the SNc, as well as in the VTA (Figure 4). All FG immunoreactive neurons (FG-ir) expressed TH. As described above, CB-ir was only found in a subpopulation of TH-ir neurons in the SNcd and in the VTA (Figure 4). However, none of the TH-ir/CB-ir neurons in SNcd and VTA showed FG labeling.

Similar results were obtained following the delivery of CTB in the putamen nucleus. All the observed CTB immunoreactive neurons (CTB-ir) were identified as TH-ir in SNcd, SNcv and VTA territories. Furthermore, all CB-ir neurons were also positive for TH. Similarly to what was observed for nigro-caudate projection neurons, none of the nigro-putaminal projection neuron did express CB (Figure 5). In other words, our tract-tracing data indicated that although the identified nigrostriatal-projecting neurons (innervating wither the caudate or the putamen nucleus) were all TH-ir, all these neurons completely lacked CB immunoreactivity.

### Expression of TH and CB in neurons giving rise to nigroextrastriatal projections

**Figure 6:**
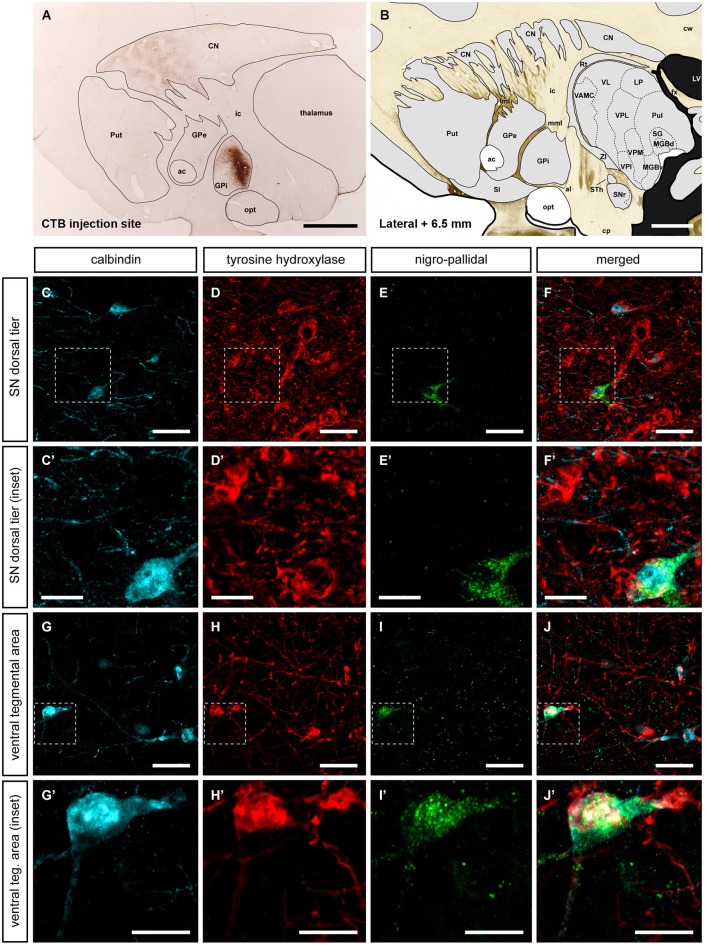
**Nigroextrastriatal neurons innervating the internal division of the globus pallidus (GPi)**. Following the delivery of the retrograde tracer CTB **(A, B)**, very few CTB-labeled neurons were found in the SNcd and in VTA **(E, E’; I, I’)**. It is worth noting that nigropallidal-projecting neurons were never found in SNcv. Even at low magnification **(panel A)**, a substantial number of CTB-labeled neurons were easily noticed in the caudate nucleus and to a lesser extent in medial territories of the putamen. At the level of the SNcd **(panels C–F’)**, all CTB-ir neurons (green channel) were also positive for both TH and CB. The same holds true when considering the VTA area, since all the VTA neurons projecting to GPi co-expressed CTB, TH and CB **(panels G–J’)**. Scale bar is 25 µm in panels **(C–F and G–J)** and 5 µm in panels **(C’–F’ and G’–J’)**.

Although the data gathered from identified nigrostriatal neurons showed that these neurons did not contain CB, the basal ganglia territories innervated by neurons co-expressing TH and CB remained to be elucidated. Accordingly, two more control primates were injected with CTB in the internal section of the globus pallidus (GPi) to further identify nigroextrastriatal neurons innervating the GPi (Figure 6A). Tracer leakage through the needle tract was not seen in any of the injected animals (Figure 6A).

The simultaneous triple stain for TH, CB and CTB showed that TH and CB co-localized in all the observed CTB-ir neurons. In other words, the observed nigro-pallidal projection neurons in the VTA and SNcd were all CB-ir/TH-ir (Figure 6).

## Discussion

CB have been proposed as a resilience factor in the DA neurons of the midbrain; the presence of this calcium binding protein could efficiently buffer Ca^+2^, therefore reducing vulnerability to mitochondrial toxins, ultimately conferring resistance to the PD-related toxins (Gerfen et al., 1985, 1987; Yamada et al., 1990; German et al., 1992; Ito et al., 1992; Ng et al., 1996; Damier et al., 1999b; Hurley et al., 2013; Yuan et al., 2013).

In the present work we have studied the content of CB within identified subpopulations of TH-ir cells located in the VTA and the SNc. Our results showed the presence of CB in TH-ir cells located in the VTA and in the SNcd, whereas CB was absent in the TH-ir cells of the SNcv. We also observed the presence of CB within the few TH-ir surviving cells of the SNcd and in the VTA from MPTP-treated monkeys. These results are in keeping with earlier reports (Gerfen et al., 1987; Yamada et al., 1990; Lavoie and Parent, 1991; Varastet et al., 1994; Damier et al., 1999a,b). However, after identifying different subpopulations of nigral efferent neurons innervating the caudate, the putamen or the internal division of the globus pallidus, we have found that nigrostriatal-projecting neurons were all CB negative, whereas co-localization of TH and CB was only found in those nigral neurons giving rise to nigroextrastriatal -nigropallidal- projections.

The highest percentage of co-localization of TH and CB was observed in the VTA (58.2%), followed by the SNcd (34.7%), similarly to previous studies in macaques reporting a co-localization rate of 43% in the VTA and 22% in the SNcd (Lavoie and Parent, 1991). In keeping with Lavoie and Parent (1991), co-localization of TH and CB was only found in the SNcd, since SNcv neurons lacked CB expression. Those results are also comparable to the TH and CB immunoreactivities observed in the human midbrain, where CB-ir cells were only found in the VTA and SNcd (Damier et al., 1999b).

As expected, the MPTP treatment produced severe dopaminergic depletion in the macaque striatum (German et al., 1988; Varastet et al., 1994; Jackson-Lewis et al., 1995; Mazloom and Smith, 2006; Rico et al., 2010), together with a gradient of neuronal loss in midbrain dopaminergic nuclei (German et al., 1988; Lavoie and Parent, 1991; Rico et al., 2010). After MPTP administration, TH-ir neurons in the SNcv are almost completely removed, followed by neurons in the SNcd, and to a lesser extent those dopaminergic neurons in the VTA. This heterogeneous pattern of cell loss properly mimicked the one observed in PD patients (German et al., 1988, 1996; Varastet et al., 1994; Liang et al., 1996; Oiwa et al., 2003; Fitzpatrick et al., 2005). Comparing control and MPTP-treated macaques, similar percentages of TH/CB co-localization were found in the VTA (58.2 vs. 62.1, respectively), whereas at the level of the SNcd, most of the surviving dopaminergic neurons expressed CB following MPTP treatment (88.6% compared to 34.7% in control animals).

Although these data supported the potential neuroprotective effect of CB against MPTP-induced dopaminergic cell degeneration, data gathered from the retrograde tracing studies conducted here envisioned a different argument by showing that nigrocaudate- and nigroputaminal-projecting TH-ir neurons did not expressed CB in the VTA and in the SNcd nuclei. In other words, TH-ir/CB-ir neurons in VTA and SNcd do not innervate the caudate/putamen. Data provided here showed that nigrostriatal-projecting neurons lacked CB, whereas those expressing CB (the most resistant ones against MPTP) were those neurons innervating the GPi through nigroextrastriatal projections. It is worth noting that TH-ir fibers are still observed in the GPi after chronic MPTP treatment (Parent et al., 1990; Lavoie and Parent, 1991; Varastet et al., 1994) and indeed earlier reports already suggested that nigroextrastriatal-projecting dopaminergic neurons are less prone to MPTP-induced degeneration (Schneider et al., 1987; Parent et al., 1990; Schneider and Dacko, 1991; Varastet et al., 1994). Nevertheless, it is worth noting that the CTB retrograde tracer deposits made here were all located in the dorsolateral postcommissural putamen and therefore the potential co-localization of CB and TH in VTA/SNcd neurons innervating striatal territories other than the dorsolateral postcommissural putamen cannot be ruled out. Furthermore, nigroextrastriatal targets other than the GPi nucleus such as the subthalamic nucleus and the external division of the globus pallidus were not investigated in this study.

## Concluding remarks

The potential role of the nigroextrastriatal system in PD pathophysiology has long been neglected (Rommelfanger and Wichmann, 2010) and indeed some studies have suggested that this system plays an important role in the early compensatory changes in PD (Obeso et al., 2004). Here we have found that midbrain dopaminergic neurons innervating extrastriatal targets were the only ones containing CB. Data reported here sustain the presence of a potential imbalance between the nigrostriatal and nigroextrastriatal systems in advanced diseases states. It seems clear that dopaminergic inputs reaching the GPi nucleus are better preserved that striatal dopaminergic innervation. Compared to the early degeneration of the nigrostriatal system, the nigroextrastriatal system is less prone to degenerate, a phenomenon that merits further research efforts in order to properly elucidate their role both in the normal and diseased basal ganglia.

## Conflict of interest statement

The authors declare that the research was conducted in the absence of any commercial or financial relationships that could be construed as a potential conflict of interest.
